# Protective effects of grape seed procyanidin on isoflurane-induced cognitive impairment in mice

**DOI:** 10.1080/13880209.2020.1730913

**Published:** 2020-03-02

**Authors:** Xiangdan Gong, Lizhi Xu, Xin Fang, Xin Zhao, Ying Du, Hao Wu, Yue Qian, Zhengliang Ma, Tianjiao Xia, Xiaoping Gu

**Affiliations:** a Department of Anesthesiology, Nanjing University Medical School Affiliated Nanjing Drum Tower Hospital, Nanjing, China; b Jiangsu Key Laboratory of Molecular Medicine, Nanjing University Medical School, Nanjing, China

**Keywords:** Cognitive dysfunction, antioxidant, p-NR2B, p-CREB

## Abstract

**Context:**

Oxidative imbalance-induced cognitive impairment is among the most urgent clinical concerns. Isoflurane has been demonstrated to impair cognitive function via an increase in oxidative stress. GSP has strong antioxidant capacities, suggesting potential cognitive benefits.

**Objective:**

This study investigates whether GSP pre-treatment can alleviate isoflurane-induced cognitive dysfunction in mice.

**Materials and methods:**

C57BL/6J mice were pre-treated with either GSP 25–100 mg/kg/d for seven days or GSP 100–400 mg/kg as a single dose before the 6 h isoflurane anaesthesia. Cognitive functioning was examined using the fear conditioning tests. The levels of SOD, p-NR2B and p-CREB in the hippocampus were also analysed.

**Results:**

Pre-treatment with either a dose of GSP 50 mg/kg/d for seven days or a single dose of GSP 200 mg/kg significantly increased the % freezing time in contextual tests on the 1st (72.18 ± 12.39% vs. 37.60 ± 8.93%; 78.27 ± 8.46% vs. 52.72 ± 2.64%), 3rd (93.80 ± 7.62% vs. 52.94 ± 14.10%; 87.65 ± 10.86% vs. 52.89 ± 1.73%) and 7th (91.36 ± 5.31% vs. 64.09 ± 14.46%; 93.78 ± 3.92% vs. 79.17 ± 1.79%) day after anaesthesia. In the hippocampus of mice exposed to isoflurane, GSP 200 mg/kg increased the total SOD activity on the 1st and 3rd day and reversed the decreased activity of the NR2B/CREB pathway.

**Discussion and conclusions:**

These findings suggest that GSP improves isoflurane-induced cognitive dysfunction by protecting against perturbing antioxidant enzyme activities and NR2B/CREB pathway. Therefore, GSP may possess a potential prophylactic role in isoflurane-induced and other oxidative stress-related cognitive decline.

## Introduction

In the recent years, acceleration in the aging population aging, has attracted enormous attention towards oxidative stress induced, age-related cognitive decline and neurodegeneration, for example, Alzheimer’s and Parkinson’s disease (Lu et al. 2014; Persson et al. 2014; Jiang et al. 2016). In addition, advances in medical care have increased the applicability of anaesthesia, beyond operations, such as painless induced delivery or endoscopy. One of the most widely used inhalational anaesthetics, isoflurane, has been shown to impair cognitive function (Lin and Zuo 2011; Zuo et al. 2018), largely caused by severe oxidative damage (Hu et al. 2011; Zhang Y et al. 2012; Miao et al. 2015; Ni et al. 2017).

Natural dietary procyanidins are bioactive food compounds, primarily present in fruits and vegetables (Pan and Ho 2008; Rodriguez-Casado 2016). Among these, grape seed procyanidin (GSP) is the most commonly used nutritional supplement. GSP exhibits superior antioxidant effect, compared to vitamin C and E, via modulation of the production of reactive oxygen species (ROS) and antioxidant enzyme activities (Kim et al. 2013; Bagchi et al. 2014).

GSP exerts protective effects on the skin, liver, heart, kidney and brain (Pataki et al. 2002; Sharma et al. 2007; Baiges et al. 2010; Kim et al. 2013; Nazima et al. 2015; Yang et al. 2017) and exhibits potential therapeutic benefits against various diseases, such as cerebral ischaemia, Alzheimer’s disease, depression and osteoarthritis (Ksiezak-Reding et al. 2012; Safwen et al. 2015; Mevel et al. 2016; Wang et al. 2018). In addition, recent research has demonstrated that procyanidins from grapes and other fruits such as blueberries can improve cognition in both young and aged subjects (Whyte et al. 2016; Haskell-Ramsay et al. 2017; Miller et al. 2018).

In this investigation, we evaluated the protective effects of GSP [2-(3,4-dihydroxyphenyl)-2-{(2-[3,4-dihydroxyphenyl]-3,4-dihydro-5,7-dihydroxy-2H-1-benzopyran-3-yl) oxy]-3,4-dihydro-2H-1-benzopyran-3,4,5,7-tetrol; CAS number: 4852-22-6] on 6 h long-term isoflurane anaesthesia-induced cognitive dysfunction. Here, the goal was to identify a potential preventive dietary therapy for anaesthesia-induced and other oxidative stress-related cognitive decline.

## Materials and methods

### Animals and GSP pre-treatment

Male C57BL/6J mice 8–12 weeks old (body weight, 20–25 g) (Model Animal Research Center of Nanjing University, Nanjing, China) were used. Water and food were provided *ad libitum*. The room temperature and humidity conditions were maintained at 23 ± 1 °C and 50–70%, respectively, under a 12 h light/dark cycle beginning at 08:00. All procedures were conducted in accordance with the applicable laws and guidelines on animal care and were approved by the Nanjing University Institutional Animal Care and Use Committee.

GSP powder was purchased from Meilun Biological Technology (Dalian, China), with the CAS number 4852-22-6. The formula of GSP is C_30_H_26_O_13_; the purity of the compound, determined using ultraviolet (UV), was over 95%. GSP powder was suspended in normal saline (NS) for gavage. To study the effect of GSP on cognitive function in mice exposed to 6 h long-term isoflurane anaesthesia, the mice were randomly assigned to NS gavage, NS gavage + 6 h isoflurane anaesthesia (NS + Ane), GSP gavage (GSP) and GSP gavage + 6 h isoflurane anaesthesia (GSP + Ane) groups. To clarify the effect of different doses and duration of GSP pre-treatment on cognitive function, in the GSP and GSP + Ane groups, the mice received, via oral gavage, either a dose of GSP 25, 50 and 100 mg/kg/d for seven days (GSP 25 × 7, *n* = 6; GSP 50 × 7, *n* = 6; GSP 100 × 7, *n* = 6; GSP 25 × 7 + Ane, *n* = 6; GSP 50 × 7 + Ane, *n* = 6; and GSP 100 × 7 + Ane, *n* = 6) or a single dose of GSP 100, 200 and 400 mg/kg (GSP 100 × 1, *n* = 6; GSP 200 × 1, *n* = 6; GSP 400 × 1, *n* = 6; GSP 100 × 1 + Ane, *n* = 6; GSP 200 × 1 + Ane, *n* = 6; and GSP 400 × 1 + Ane, *n* = 6) before the administration of isoflurane anaesthesia. The NS and NS + Ane groups in each test simultaneously received an oral gavage of NS in the same volume (NS × 7, *n* = 6; NS × 1, *n* = 6; NS × 7 + Ane, *n* = 6; NS × 1 + Ane, *n* = 6). For all groups, the administration time was maintained as 20:00.

### Isoflurane anaesthesia

In the NS + Ane and GSP + Ane isoflurane group, mice were placed in a chamber with 4% isoflurane carried by 100% O_2_ for anaesthesia induction 2 h after the last GSP administration, followed by exposure to 1.2% isoflurane in 100% O_2_ for 6 h. During the anaesthesia, body temperature and respiratory frequency were monitored as vital signs and heat sheets were used to maintain the body temperature of the mice at 36.8 ± 1 °C.

### Fear condition test

Fear condition tests were performed on the 1st, 3rd, 7th and 14th day. Sequentially, mice were placed in a chamber (25 × 25 × 25 cm^3^) (Panlab/Harvard Apparatus, Barcelona, Spain) for 5 min of habituation and free exploration of the context. Next, they were subjected to a conditioned stimulus (CS) (28 s, 4 kHz, 90 dB), followed by a foot-shock signal (2 s, 0. 8 mA) from the floor in the chamber. The chambers were cleaned with 75% alcohol between each session. Both contextual memory evaluation and cue memory evaluation were performed the following day. For contextual tests, mice were placed in the same chamber for 5 min without foot shock. For the cue tests, the chambers were modified to have an altered appearance, and the mice were exposed to the same CS cue without the foot-shock. The freezing state was recorded using the PACKWIN system (Panlab/Harvard Apparatus, Barcelona, Spain). Percent freezing time = freezing time/phase time × 100%.

### Tissue collection

Mice were anaesthetized by isoflurane and promptly killed after fear condition tests on the 1st, 3rd and 7th day after anaesthesia. The brain was quickly separated, dissected and the hippocampus was frozen by liquid nitrogen and stored at −80 °C for superoxide dismutase (SOD) activity and protein analysis.

### Superoxide dismutase activity analysis

The total SOD activity was detected using a SOD assay kit (Beyotime, Shanghai, China) in accordance with the manufacturer’s guidelines. The activity levels of SOD were modified to U/mg of protein.

### Western blot

The samples were washed with pre-cooled PBS before being lysed with RIPA lysis buffer (Beyotime, Shanghai, China) containing a mixture of protease and phosphatase inhibitors (Pierce, Rockford, IL). The protein concentration was measured using the bicinchoninic acid (BCA) protein assay kit (Pierce, Rockford, IL). Protein samples (25 µg for each lane) were separated using SDS-PAGE gels and transferred to polyvinylidene (PVDF) membranes (Bio-Rad Laboratories, Hercules, CA). Next, the membranes were then blocked with 5% BSA at room temperature for 2 h and incubated with a diluted primary antibody (p-CREB) (phospho Ser133), 1:2500, rabbit monoclonal antibody (Abcam, Cambridge, UK); p-NR2B (phospho Y1472), 1:5000, rabbit polyclonal antibody (Abcam, Cambridge, UK) and for 1 h at room temperature with an HRP-conjugated secondary antibody, 1:20,000 (Beyotime, Shanghai, China). The blots were detected using ECL solution (Pierce, Rockford, IL) and exposed in the Chemiluminescence imaging analysis system (Tanon, Shanghai, China) for 1–10 min.

### Statistical analysis

Statistical analyses were performed using GraphPad Prism version 6.00 (GraphPad software, La Jolla, CA) and SPSS version 22.0.0 (IBM, Armonk, NY). To perform a comparison of all groups in both behavioural and biochemical analyses, one-way ANOVA, followed by Bonferroni’s comparison, was used. Data are presented as the mean ± SD. A *p* Value less than 0.05 was considered statistically significant.

## Results

### Six-hour long-term isoflurane anaesthesia impaired contextual memory in mice

**Figure 1.**
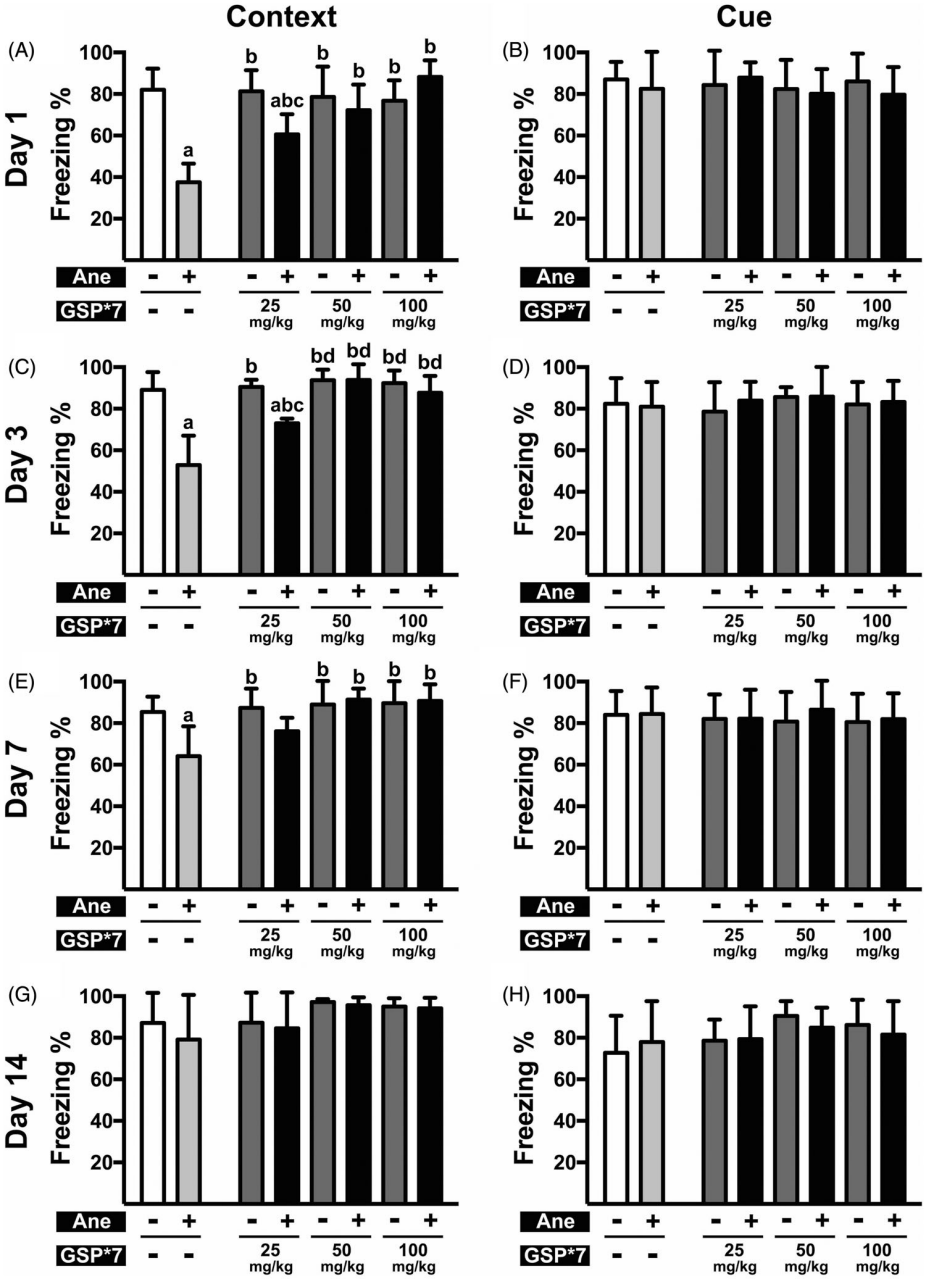
Cognitive function post anaesthesia in mice pre-treated with various doses of GSP for 7 days. (A, C, E, G) Contextual memory in the NS × 7 (Ane –, GSP × 7 –), NS × 7 + Ane (Ane +, GSP × 7 –), GSP × 7 (Ane –, GSP × 7 +) and GSP × 7 + Ane (Ane +, GSP × 7 +) groups on the 1st (A), 3rd (C), 7th (E) day and 14th (G) day after anaesthesia. (B, D, F, H) Cue memory in the NS × 7, NS × 7 + Ane, GSP × 7 and GSP × 7 + Ane groups on the 1st (B), 3rd (D), 7th (F) and 14th (H) day after anaesthesia. Values are expressed as the mean ± SD (*n* = 6). ^a^*p* < 0.05, compared to the NS × 7 group; ^b^*p* < 0.05, as compared to the NS × 7 + Ane group; ^c^*p* < 0.05, compared to the GSP25 × 7 group; ^d^*p* < 0.05, compared to the GSP25 × 7 + Ane group.

**Figure 2.**
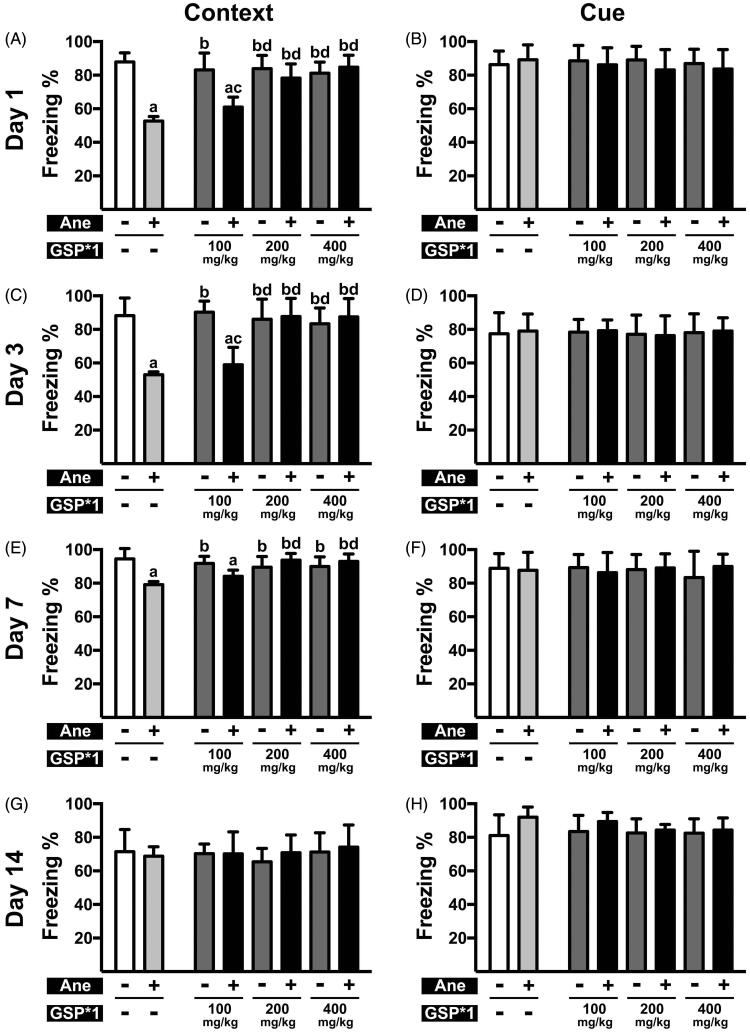
Cognitive function post anaesthesia in mice pre-treated with a single doses of GSP at varying concentrations. (A, C, E, G) Contextual memory in NS × 1 (Ane –, GSP × 1 –), NS × 1 + Ane (Ane +, GSP × 1 –), GSP × 1 (Ane –, GSP × 1 +) and GSP × 1 + Ane (Ane +, GSP × 1 +) groups on the 1st (A), 3rd (C), 7th (E) day and 14th (G) day after anaesthesia. (B, D, F, H) Cue memory in NS × 1, NS × 1 + Ane, GSP × 1, GSP × 1 + Ane groups at 1st (B), 3rd (D) 7th (F) and 14th (H) day after anaesthesia. Values are expressed as the mean ± SD (*n* = 6). ^a^*p*< 0.05, compared to the NS × 1 group; ^b^*p* < 0.05, as compared to the NS × 1 + Ane group; ^c^*p* < 0.05, compared to the GSP100 × 1 group; ^d^*p* < 0.05, compared to the GSP100 × 1 + Ane group.

Previously, our research demonstrated that spatial memory, detected by Intellicage, was significantly impaired after 6 h long-term isoflurane anaesthesia in mice (Xia et al. 2016). To determine the influence of 6 h long-term isoflurane anaesthesia on contextual and cue memory, we used the fear conditioning test. We observed that, in contextual tests, the percent freezing time in mice pre-treated with NS for seven days prior to anaesthesia was significantly reduced on the 1st (37.60 ± 8.93% vs. 82.02 ± 10.25%, *n* = 6, *p* < 0.001), 3rd (52.94 ± 14.10% vs. 89.06 ± 8.61%, *n* = 6, *p* < 0.001) and 7th day (64.09 ± 14.46% vs. 85.45 ± 7.26%, *n* = 6, *p* = 0.01) after anaesthesia compared with the NS × 7 group. The contextual memory in mice pre-treated with NS as a single oral gavage was essentially the same on the 1st (52.72 ± 2.64% vs. 87.88 ± 5.47%, *n* = 6, *p* < 0.001), 3rd (52.89 ± 1.73% vs. 88.22 ± 10.44%, *n* = 6, *p* < 0.001) and 7th (79.17 ± 1.79% vs. 94.45 ± 6.21%, *n* = 6, *p* < 0.001) day after anaesthesia, whereas cue memory was not affected (Figures 1 and 2). These results suggest that 6 h long-term isoflurane anaesthesia impaired cognitive function, and that NS pre-treatment has no significant influence on cognitive performance.

### GSP pre-treatment reversed isoflurane anaesthesia-induced cognitive dysfunction

As shown in Figure 1, contextual memory in the GSP 25 × 7 + Ane group demonstrated a significant increase, compared to the NS × 7 + Ane group, on the 1st and 3rd day after anaesthesia (60.61 ± 9.66% vs. 37.60 ± 8.93%, *n* = 6, *p*= 0.016; 72.99 ± 2.25% vs. 52.94 ± 14.10%, *n* = 6, *p*= 0.002), but not on the 7th day (76.15 ± 6.47% vs. 64.09 ± 14.46%, *n* = 6, *p*= 0.376). However, 50 mg/kg/d or more, for seven days, caused a greater improvement in isoflurane-induced cognitive dysfunction, as contextual memory performances of the GSP 50 × 7 + Ane group and the GSP 100 × 7 + Ane group were significantly improved, compared to the NS × 7 + Ane group on the 1st (72.18 ± 12.39% vs. 37.60 ± 8.93%, *n* = 6, *p* < 0.001; 88.19 ± 8.05% vs. 37.60 ± 8.93%, *n* = 6, *p* < 0.001), 3rd (93.80 ± 7.62% vs. 52.94 ± 14.10%, *n* = 6, *p* < 0.001; 87.71 ± 8.11% vs. 52.94 ± 14.10%, *n* = 6, *p* < 0.001) and 7th (91.36 ± 5.31% vs. 64.09 ± 14.46%, *n* = 6, *p* < 0.001; 90.71 ± 8.04% vs. 64.09 ± 14.46%, *n* = 6, *p* < 0.001) day after anaesthesia. In addition, contextual memory in all of the GSP × 7 groups was not affected, moreover, no significant changes were observed in cue memory for all treatments.

Further, we examined whether a single dose of GSP is effective (Figure 2); here, we observed that a single dose greater than 200 mg/kg of GSP, prior to anaesthesia, exerted a comparable protective effect, as seen with GSP 50 × 7 pre-treatment on the 1st (78.27 ± 8.46% vs. 52.72 ± 2.64%, *n* = 6, *p* < 0.001; 84.72 ± 7.21% vs. 52.72 ± 2.64%, *n* = 6, *p* < 0.001), 3rd (87.65 ± 10.86% vs. 52.89 ± 1.73%, *n* = 6, *p* < 0.001; 87.50 ± 10.91% vs. 52.89 ± 1.73%, *n* = 6, *p* < 0.001) and 7th (93.78 ± 3.92% vs. 79.17 ± 1.79%, *n* = 6, *p* < 0.001; 93.03 ± 4.38% vs. 79.17 ± 1.79%, *n* = 6, *p* < 0.001) day; in contrast, the GSP 100 × 1 + Ane group demonstrated no significant difference compared to the NS × 1 + Ane group on the 1st (60.95 ± 5.94% vs. 52.72 ± 2.64%, *n* = 6, N.S.), 3rd (58.99 ± 10.28% vs. 52.89 ± 1.73%, *n* = 6, N.S.) and 7th (84.09 ± 3.66% vs. 79.17 ± 1.79%, N.S.) day. All pre-treatments displayed no effect on cue memory.

### SOD activity and the NR2B/CREB pathway were involved in the protective effect of GSP

**Figure 3.**
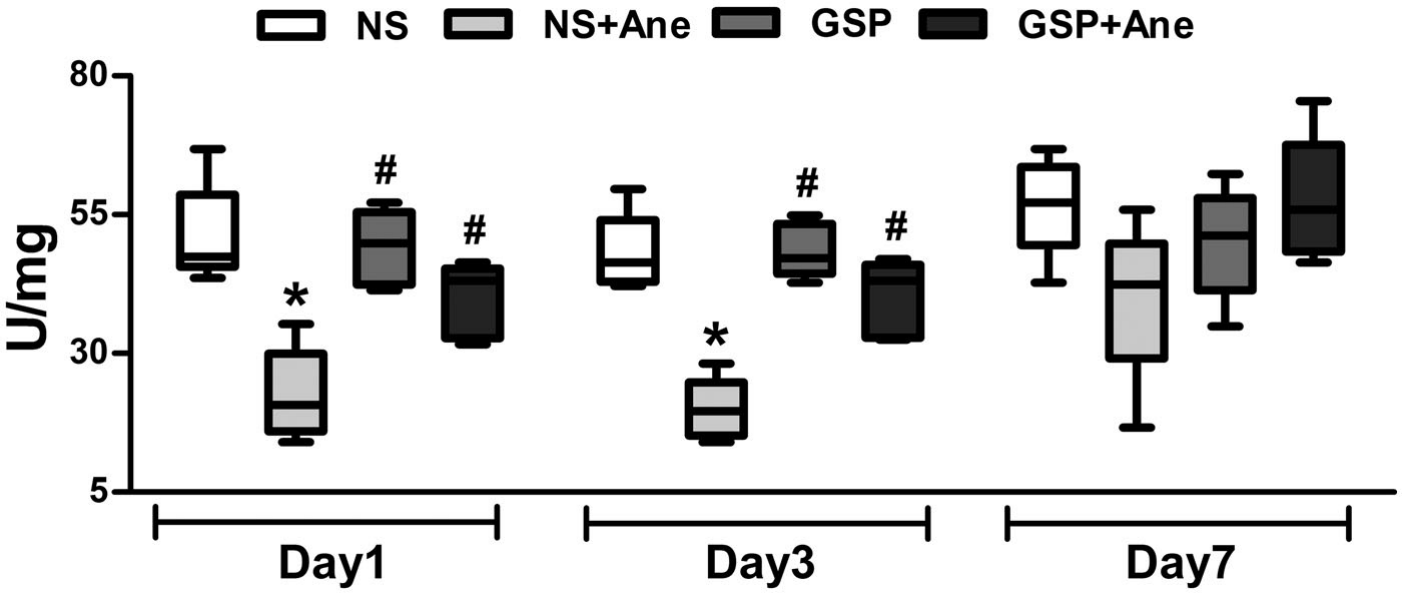
SOD activity post 6 h long-term isoflurane anaesthesia and GSP pre-treatment with a single dose. SOD activity was significantly decreased after 6 h long-term isoflurane anaesthesia on the 1st and 3rd day after anaesthesia. A single dose of GSP 200 mg/kg pre-treatment reversed the impairment of SOD activity. Values are expressed as the mean ± SD (*n* = 5). *p< 0.05, compared to the NS × 1 group, ^#^*p* < 0.05, compared to the NS × 1 + Ane group.

To investigate the mechanism underlying the protective effect of GSP, we analysed oxidative stress-related SOD activity and the cognition-related NR2B/CREB pathway in the hippocampus of mice treated with a single dose of GSP 200 mg/kg and anaesthesia. Compared to the NS × 1 group, our results demonstrated that the total SOD activity in the NS × 1 + Ane group was also significantly decreased on the 1st (22.61 U/mg vs. 51.24 U/mg, *n* = 5, *p* < 0.001) and 3rd (19.91 5 U/mg vs. 48.05 U/mg, *n* = 5, *p* < 0.001) day after anaesthesia. Furthermore, GSP 200 mg/kg, as a single dose, significantly increased the total SOD activity on the 1st (40.38 U/mg vs. 22.61 U/mg, *n* = 5, *p* < 0.001) and 3rd (40.12 U/mg vs. 19.91 U/mg, *n* = 5, *p* < 0.001) day (Figure 3).

**Figure 4.**
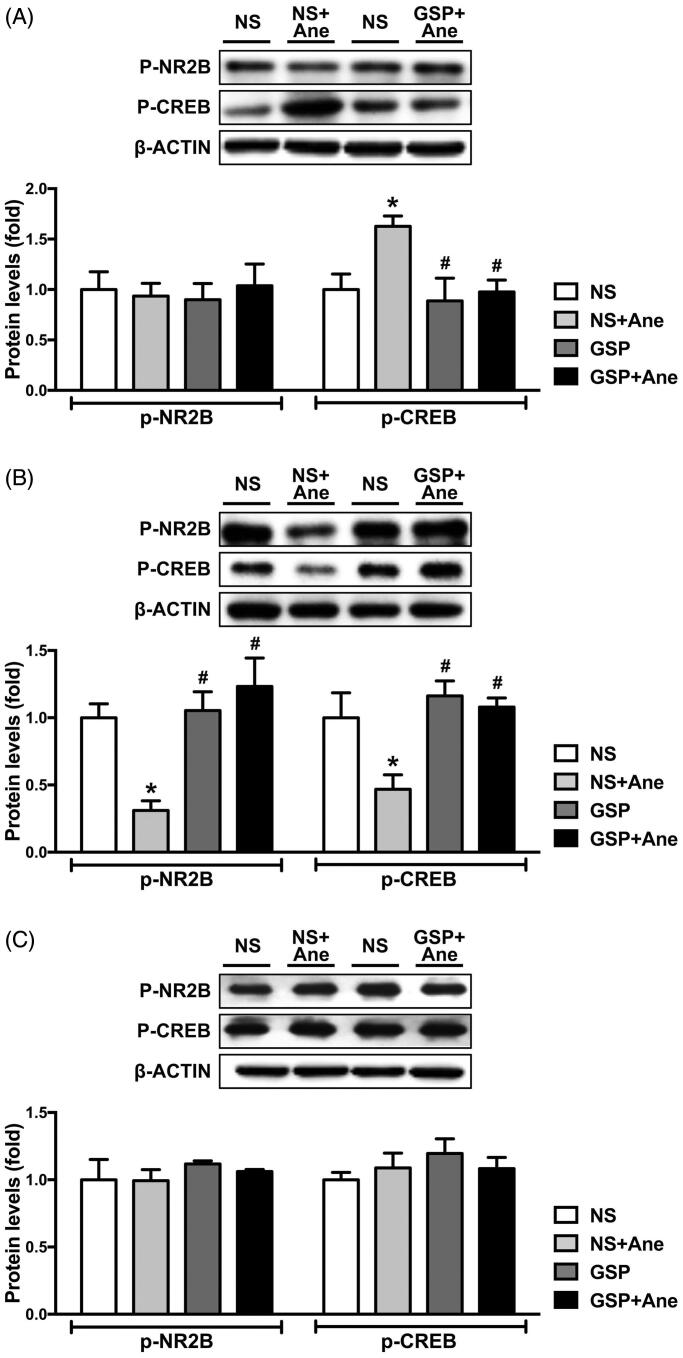
Effects of a single dose GSP 200 mg/kg on NR2B and CREB phosphorylation status on the 1st (A), 3rd (B) and 7th (C) day after anaesthesia. p-NR2B protein levels in the NS × 1 + Ane group significantly decreased on the 3rd day (B) after anaesthesia; p-CREB protein levels in anaesthesia group was significantly increased on the 1st day (A) after anaesthesia and significantly decreased on the 3rd day (B). All these changes were reserved after a single dose of GSP 200 mg/kg. Representative blots of each protein and statistical analysis of the relative protein expression are shown. Values are expressed as the mean ± SD (*n* = 3). *p< 0.05, compared to the NS × 1 group; ^#^*p* < 0.05, compared to the NS × 1 + Ane group.

We next explored the phosphorylation levels of NR2B and CREB in the hippocampus (Figure 4). Consistent with our previous results, the phosphorylation status of CREB was significantly increased on the 1st day after anaesthesia, compared to the NS × 1 group (Figure 4(A)). On the 3rd day, the levels of both NR2B phosphorylation and CREB phosphorylation in the NS × 1 + Ane group were significantly decreased (Figure 4(B)). Furthermore, GSP 200 mg/kg pre-treatment significantly reversed the changes of p-NR2B and p-CREB levels on the 1st and 3rd day after anaesthesia (Figure 4(A,B)). On the 7th day post anaesthesia, the p-NR2B and p-CREB levels demonstrated no differences across all groups (Figure 4(C)).

## Discussion

In the current study, we evaluated the implications of isoflurane and GSP interaction on cognitive function. Our results demonstrated that GSP pre-treatment improved 6 h long-term isoflurane anaesthesia-induced cognitive impairment. In addition, the total SOD activity and the NR2B/CREB pathway were demonstrated to be involved in the protective effects of GSP. These findings suggest that GSP could represent a potential preventive treatment for isoflurane anaesthesia-induced cognitive impairment.

Previous studies showed that, in Western countries, the prevalence of dementia in people over 60 is greater than 5%, and nearly doubles every 5 years after the age of 65, comprising about 50 million individuals worldwide (Plassman et al. 2007; Jonsson et al. 2012). With the growing increase in the aging population, the development of potential therapies for neurodegenerative and age-induced cognitive dysfunctions has become an urgent clinical challenge. As oxidative stress is important in the pathogenesis of age-related cognitive decline and neurodegenerative diseases, such as Alzheimer’s and Parkinson’s disease (Kemppainen et al. 2015; Melief et al. 2015), it has become an anticipated target.

Oxidative stress represents the existence of an imbalance between the generation of ROS and the ability to neutralize ROS by the antioxidants, such as antioxidant enzymes and non-enzymatic antioxidant factors (Gonzalez-Dominguez et al. 2014). If cellular antioxidants fail to scavenge ROS, oxidative damage ensues, leading to cytotoxicity and resulting in protein collapse, enzyme failure and lipid destruction (Lee et al. 2012). Furthermore, this could result in the destruction of the neuronal tissue, causing cognitive impairment (Kojima et al. 2013; Rodrigues et al. 2017).

Isoflurane, one of the most widely used inhalational anaesthetics, is extensively used beyond surgical interventions, including painless induced delivery or endoscopy. Previously, results from our lab and other studies demonstrated that cognitive functions were significantly impaired following isoflurane anaesthesia in both human and mice (Zhang B et al. 2012; Xia et al. 2016; Fang et al. 2017; Ni et al. 2017; Zuo et al. 2018). It has also been reported that isoflurane causes severe oxidative stress by impairing antioxidant enzymes, inducing mitochondrial dysfunction and increasing the levels of ROS (Zhang Y et al. 2012).

Natural products contain several active ingredients that have proven to be effective in the prevention or treatment of diseases. In recent years, phytochemicals have received enormous attention due to their strong antioxidant, anti-inflammatory and anticancer activities. GSP is commonly used as a nutritional supplement (Ferruzzi et al. 2009) and has been attributed as a potential health food ingredient according to the generally recognized as safe (GRAS) certification from USA FDA (Bomser et al. 1999). Previous research has demonstrated that procyanidins from a variety plants and several other kinds of antioxidants have neuroprotective effects in various central nervous system disease models, including age-related cognitive dysfunction, traumatic brain injury and psychotropic drug toxicity (Zhang et al. 2004; Xu et al. 2010; Mao et al. 2015; Gong et al. 2016). In the recent years, clinical studies on berry fruits, including strawberries, blueberries, black currants and grapes, which represent a rich source of flavonoids, have reported benefits on cognitive function, such attention, executive function, short-term memory and long-term memory (Broadbent et al. 2004; Whyte et al. 2016). However, whether antioxidants, such as GSP, have protective effects on oxidative stress-induced cognitive dysfunction remains ambiguous.

In this study, GSP was used to assess their antioxidant effects in a mice model of 6 h isoflurane anaesthesia-induced cognitive dysfunction. Different doses of GSP were administered to identify if the protection depended on dose and, consequently, evaluate the most effective dose. As the maximum ingestion of procyanidins in individuals from different countries can reach 400–450 mg/day, a dose of 25–100 mg/kg was used in mice as a medium dose, which can be obtained through healthy eating habit or, partly, through the intake of nutritional supplements (Zamora-Ros et al. 2010; Knaze et al. 2012). This conversion between human and mice doses has been normalized by the body surface area (BSA)-based dose calculation (Reagan-Shaw et al. 2008). In addition, as the LD_50_ of procyanidins is higher than 5000 mg/kg in rats and no significant toxicity has been reported at present, 400 mg/kg as a single dose is considered safe and convenient for clinical practice (Ferruzzi et al. 2009). We demonstrated that GSP improved isoflurane-induced cognitive decline in mice. Our data suggest that GSP at a dose greater than 200 mg/kg as a single dose or 50 mg/kg/d for seven days could mitigate the neurobehavioural impairment induced by isoflurane anaesthesia.

SOD is one of the oxygen radical scavengers natively existing in organisms, the activity of SOD represents the ability to scavenge free radicals. As a strong antioxidant, GSP demonstrated no significant effect on the normal activity of antioxidant enzymes; however, it can maintain antioxidant enzyme activities close to normal levels when another treatment suppresses these enzymes (Karthikeyan et al. 2007). In this study, we detected a decrease in the total SOD activity on the 1st and 3rd day after anaesthesia and both the GSP 200 × 1 group and GSP 200 × 1 + Ane group exhibited no significant difference compared to the NS × 1 group.

NR2B is required for the induction of long-term depression, which is considered to underlie learning and memory processes. Moreover, calcium entry through the extrasynaptic NMDA receptors can activate the CREB pathway, which also plays an essential role in memory formation (Martel et al. 2012). In the current study, we observed that phosphorylation of NR2B and CREB was impaired on the 3rd day after anaesthesia, whereas GSP pre-treatment can significantly increase the p-NR2B and p-CREB levels.

## Conclusions

Our findings demonstrated the potential protective effects of GSP pre-treatment on long-term isoflurane anaesthesia-induced cognitive dysfunction in mice. These protective effects of GSP are possibly associated with enhanced antioxidant enzyme activities, and stabilized NR2B/CREB pathway. Furthermore, our results suggested that sufficient intake of antioxidants, such as GSP, or a balanced diet may help avert isoflurane anaesthesia-induced or other oxidative stress-related cognitive dysfunctions.
